# Effects of the Surgical Ligation of the Ureter in Different Locations on the Kidney over Time in the Rat Model

**DOI:** 10.1155/2024/6611081

**Published:** 2024-06-06

**Authors:** Abdolreza Mohammadi, Leila Zareian Baghdadabad, Parisa Zahmatkesh, Hedieh Moradi Tabriz, Alireza Khajavi, Gholamreza Mesbah, Parsa Nikoofar, Seyed Mohammad Kazem Aghamir

**Affiliations:** ^1^Urology Research Center, Tehran University of Medical Sciences, Tehran, Iran; ^2^Department of Pathology, Sina Hospital, Tehran University of Medical Sciences, Tehran, Iran; ^3^Student Research Committee, Faculty of Paramedical Sciences, Shahid Beheshti University of Medical Sciences, Tehran, Iran; ^4^Department of Urology, Thunder Bay Regional Health Research Institute, Thunder Bay, Ontario, Canada

## Abstract

**Purpose:**

To evaluate the effects of the surgical ligation of the ureter in different locations on the kidney over time in the rat model.

**Methods:**

A total of 155 rats were enrolled and randomly divided into the case (*n* = 150) and control (*n* = 5) groups. The case group included three separate groups (fifty rats in each group) that underwent surgical ureteral ligation at the proximal, middle, and distal ureter. The laboratory tests, and tumor necrosis factor *α* (TNF-*α*), were measured in groups. The pathological evaluation for glomerular changes, tubular dilation, interstitial fibrosis, and interstitial infiltration of the inflammatory cells following the obstruction was performed (severity of tubular atrophy categorized too mild (+), moderate (++), and severe (+++)). To compare the continuous variables between the groups and between the measurement times, the analysis of variance (ANOVA) was used.

**Results:**

Our results revealed that the creatinine four weeks after the obstruction was significantly higher in the proximal group obstruction (*p* value: 0.046). The three groups had no significant differences regarding urine creatinine, serum sodium, and serum TNF (*p* value: 0.261). Obstruction did not change the glomerular morphology in three intervention groups after six weeks. The commencing of severe tubular atrophy in proximal, middle, and distal ureteral obstruction was at weeks three, four, and six, respectively.

**Conclusion:**

The location of ureteral obstruction is also crucial in deciding to intervene to relieve the complete ureteral obstruction. Severe tubular damage occurs in weeks three, four, and six in proximal, middle, and distal ureteral obstruction, respectively.

## 1. Introduction

Ureteral obstruction due to the ureteral stone could result in renal damage if left untreated for a limited time. In the acute phase of the unilateral ureteral obstruction (UUO), the early changes are an increase in the hydrostatic pressure proximal to the obstructed ureter and a decrease in the estimated glomerular filtration (e-GFR) and renal blood flow (RBF) that are reversible if the obstruction is relieved [1]. The exact time of intervention to prevent permanent renal injury is controversial. However, many studies declared that complete renal loss could occur if obstruction exceeds six weeks [2, 3]. The European Association of Urology (EAU) and American Urological Associations (AUA) guidelines recommend a two- to six-week range for intervention [4, 5]. As cited in the literature, the primary determinants of renal damage are the severity of the obstruction (complete or incomplete) and the duration of the obstruction. We hypothesized that proximal obstructions might produce more hydrostatic pressure to the kidney than distal obstruction, so the location of the obstruction and its role in determining these time limits is neglected and may have a role in deciding the proper time for the intervention. Accordingly, we designed an animal study to evaluate the impact of different levels of ureteral obstruction by surgical ligation of the ureter on the severity of renal damage in succeeding weeks of obstruction. These findings will help propose a new definition for the time limit to intervention in ureteral obstruction according to the location of the obstruction.

## 2. Materials and Methods

### 2.1. Animal Design

In this case-control study, one hundred fifty-five adult Wistar male rats weighing 200–220 g (8–10 weeks old) were enrolled. The study is reported by ARRIVE guidelines. This study was approved by the Ethics Committee of the Tehran University of Medical Sciences (IR.TUMS.SINAHOSPITAL.REC.139). They were randomly divided into the case (*n* = 150) and control (*n* = 5) groups. The case group included the three separated groups (fifty rats in each group) that underwent surgical ureteral ligation in the proximal, middle, and distal ureter. The main idea of ureteral ligation was produced based on the ureteral ligation of different time intervals. However, we designed our study based on different locations of ureteral ligation [6]. The rats were accommodated in the animal laboratory for two weeks to adapt to the environment in the separate cages under calm conditions with minimal stress and receiving 12 hours of light and 12 hours of darkness at 22°C, with a humidity of 55−50%. Two ventilators and an exhaust system were installed on either side of the rat cages. Before the intervention, the basic laboratory tests, including the urea, creatinine, and electrolytes, were performed in both case and control groups.

### 2.2. Surgical Procedure

The Wistar male rats were anesthetized with intraperitoneal ketamine injection (80 mg/kg) [7]. The lower midline incision was performed to access the peritoneal cavity. After the lateral dissection, the kidney was exposed and the ureter was released from the ureteropelvic junction to the distal part of the ureter at the ureterovesical junction. The intraperitoneal organ is retracted by a small retractor away from the ureter. The experienced veterinarian monitors the vital signs during the procedure, such as respiratory and pulse rates. In the first intervention group (fifty rats named K1), the proximal ureter, upper to the sacral brim, was ligated using the Monocryl suture 3−0 to produce a complete obstruction in the ureteral lumen. In group two (fifty rats named K2), the ureter was ligated in the middle part (at the level of the sacrum) in the same fashion. In the third intervention group (K3), the ureter was ligated in the distal part (under the sacrum; near the ureterovesical junction) to create a complete ureteral obstruction. The peritoneum layer was closed with 4−0 chromic sutures, and the abdominal layers, including the fascia, were approached by the 4−0 Monocryl sutures. In the control sham group (five rats), the midline incision was performed and the ureter was explored in the same manner as in the intervention groups. Without further manipulation of the ureter, the abdominal incision was closed, similar to the case groups. The rats were evaluated daily by an expert team of veterinarians for the infection symptoms. All rats were standardized to have the same amount of fluid and food postoperatively.

### 2.3. Laboratory Tests

The laboratory tests include urine creatinine (Urine Cr), serum creatinine (Cr), sodium (Na), and potassium (K). Also, due to the pivotal role of tumor necrosis factor *α* (TNF-*α*) in the production of interstitial fibrosis, we checked this inflammatory factor in three intervention groups weekly.

### 2.4. Evaluation of the Renal and Ureteral Pathology

Pathological evaluation of changes in the ureter and renal units following the obstruction was performed by an experienced pathologist in three intervention groups at weekly intervals (one, two, three, four, and six) after sacrificing the rats. The evaluation included four main domains: glomerular changes, tubular dilation, interstitial fibrosis, and interstitial infiltration of the inflammatory cells. Changes detected in the microscopic field (×400 magnification) evaluated the glomerular morphologic changes. The tubular dilation and interstitial fibrosis were stated according to a qualitative score: negative, + mild, ++ moderate, and +++ severe. Interstitial fibrosis is declared a percentage of the inflammatory cells in microscopic fields. After sacrificing rats in the mentioned weeks, the gross appearance of kidneys and ureters was documented by using a digital camera. The daily temperature and weight changes before and after ureteral ligation were recorded in all three groups [8, 9].

### 2.5. Imaging of the Obstructed Kidney and Ureter

We achieved an expert veterinarian radiologist's ultrasound imaging of the kidneys and ureters. The Toshiba Nemio 30 ultrasound probe (9.5 Megahertz) was used to evaluate the preoperative condition of the urinary system, the severity of hydronephrosis, and changes in renal cortical thickness at weekly intervals after the intervention.

### 2.6. Statistical Analysis

The sample consisted of three active groups and one control. In each group, 50 rats were recruited and then ten mice were sacrificed in each one of the times 1, 2, 3, 4, and 6. The continuous variables are reported using the mean (standard deviation (SD)). There were all values in the data. To compare the continuous variables between the groups and between the measurement times, the analysis of variance (ANOVA) was used. The data were analyzed using Stata (ver. 13). The significance level was chosen as 0.05.

## 3. Results

The postoperative course was uneventful for all rats. There were no complications related to the surgical ligation. The continuous variables of laboratory tests are reported in Table 1, using the median (IQR) and comparing the groups and measurement times. The *p* values were obtained from the nonparametric test for comparison of the medians. The three groups had no significant differences regarding urine Cr, serum Na, and serum TNF. The proximal, middle, and distal ureteral obstructions are stated as groups 1, 2, and 3, respectively. Table 1 indicates considerable differences between the groups in mean change in serum Cr at four weeks after obstruction, as this parameter was significantly higher in the proximal obstruction group (*p* value: 0.046), but this parameter returned to the baseline after six weeks.

**Figure 1:**
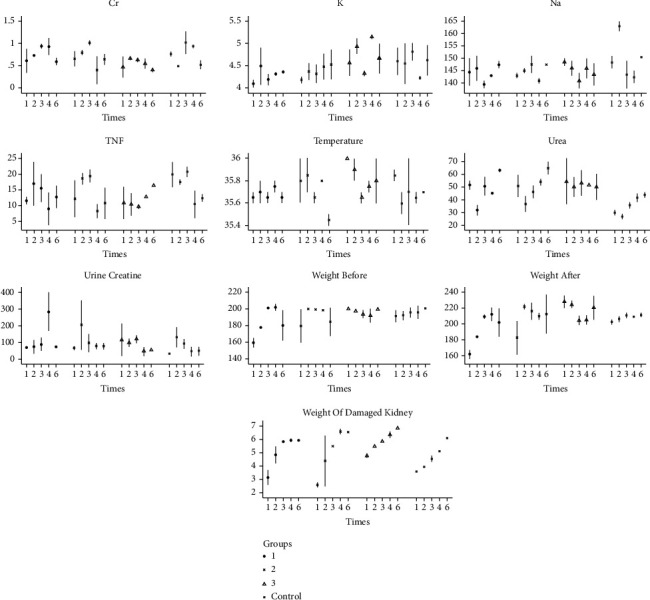
Laboratory findings in three intervention groups.

Serum K (at week 1), the weight of kidneys after obstruction (at week 2), and the weight of the damaged kidney (at week 6) was all significantly different in the distal ureteral obstruction group (*p* value: 0.046).  Figure 1 also depicts the bar chart of these findings in the three interventional groups.

### 3.1. Pathologic Findings

**Figure 2:**
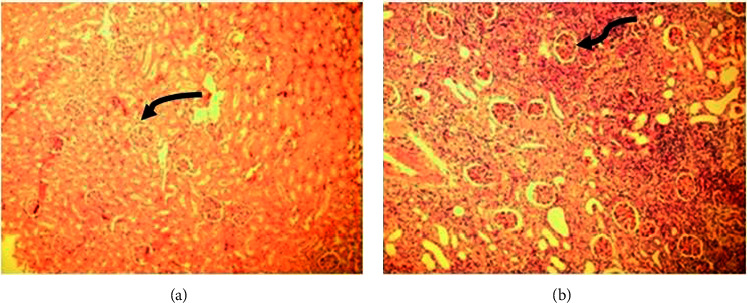
Glomerular morphology in proximal ureteral obstruction: (a) magnitude 100 and (b) magnitude 200.

The pathologic evaluation of the first group with complete proximal ureteral obstruction (K1 group) is demonstrated in Figure 2. Figures 2(a) and 2(b) demonstrate the glomerular morphology at one and six weeks after obstruction, respectively. There were no changes in glomerular morphology (solid arrows).

The other pathologic changes, including tubular atrophy, interstitial fibrosis, and interstitial inflammation, were also evaluated and summarized in Table 2. The findings revealed that the severe tubular atrophy would start at week three after unilateral ureteral obstruction (UUO).

**Figure 3:**
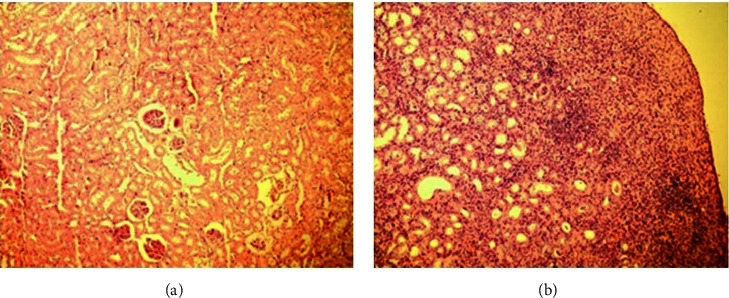
Glomerular morphology in midureteral obstruction.

Figures 3(a) and 3(b) demonstrate the glomerular morphology in weeks one and six after the midureteral obstruction, respectively. Similar to the first group, there were no changes in glomerular morphology.

Table 3 demonstrates that severe tubulopathy occurred after four weeks of obstruction in this group.

**Figure 4:**
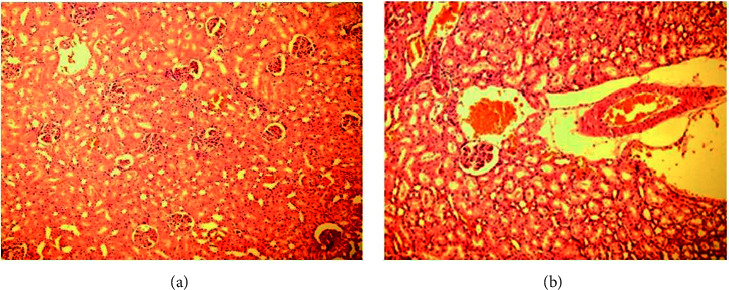
Glomerular morphology in distal ureteral obstruction: (a) magnitude 100 and (b) magnitude 200.

Figures 4(a) and 4(b) also demonstrate no changes in glomerular morphology after six weeks of obstruction in the third group (distal ureteral obstruction).

Table 4 shows that the extreme tubular atrophy started in week six after UUO in the distal ureter obstruction group.

The percentage of infiltration by inflammatory cells in the interstitium was not significantly different between the three intervention groups.

**Figure 5:**
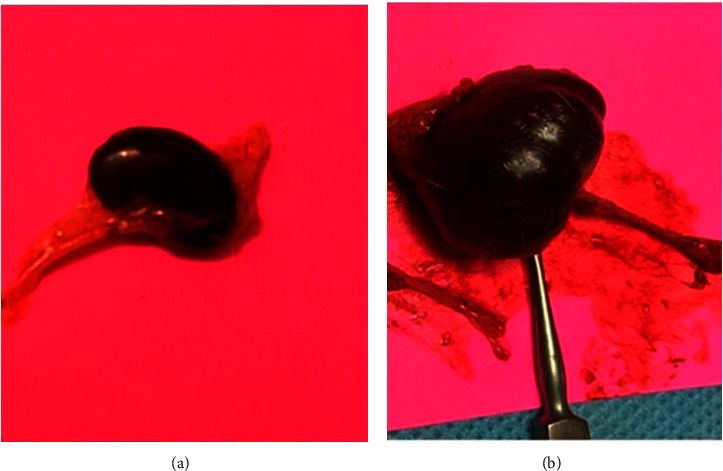
The gross shape of the kidney and ureter; proximal obstruction (a) and distal ureteral obstruction (b).

Figures 5(a) and 5(b) show the differences between the gross appearance of the kidney and ureter after six weeks of the ureteral obstruction at the distal and proximal ureter, respectively.

The ultrasound imaging of the kidney and ureter was performed in three intervention groups at weekly intervals. As for the gross morphologic changes, the ultrasound showed that the more proximal the obstruction was induced, it was associated with more severe hydronephrosis, the ultrasound imaging and pathologic data, and is attached to supplementary file 1.

## 4. Discussion

Urinary stone disease is a complex phenomenon with a multifactorial etiology, with varying incidence in different continents and climates (5–20% in Asia, 5–9% in Europe, and 7–13% in North America) [10, 11]. The ureteral calculi are among the most frequent causes of emergency department visits. Most stones with sizes less than 5 mm will usually pass spontaneously with conservative treatments, including hydration, analgesic, and alpha-blockers. In case of conservative measure failure, further therapeutic options to be offered are shockwave lithotripsy (SWL), transurethral ureterolithotripsy (TUL), and percutaneous nephrolithotomy (PCNL) [12–15]. The severity of the obstruction is not related to the size of the stone; sometimes, a small stone might result in complete obstruction of the ureter. Current literature emphasizes the stone diameter as the single determinant of optimal stone management. However, the degree of obstruction (complete vs. incomplete) and time of the obstruction are the other important factors [16]. Early ureteroscopic intervention is indicated in patients with renal failure, refractory pain, single kidney, and a large burden of stones with a low probability of spontaneous passage [5, 17, 18].

The effect of complete unilateral ureteral obstruction (UUO) was studied first time by Kerr et al.; in this animal study, they evaluated the impact of complete ureteral obstruction in the weekly intervals for 1, 2, 3, and 4 weeks on renal function and also the proportion of regained renal function following the release of the obstruction. They measured the glomerular filtration rate (GFR) initially and after the release of obstruction. Their results revealed that the relief of ureteral obstruction after one week was associated with the highest improvement in GFR. After four weeks of obstruction, there was a minimal improvement in GFR [6].

Klahr conducted a comprehensive study on the pathophysiology of renal function impairment in obstruction. The study observed two models of obstruction, namely, unilateral or bilateral ureteral ligation. The decrease in whole kidney GFR after the release of obstruction is due to two factors: a decrease in the single-nephron glomerular filtration rate (SNGFR) and a decrease in the number of filtering nephrons. The reduction in SNGFR is caused by a decrease in plasma flow per nephron, a decrease in net hydrostatic pressure, and a decrease in the filtration coefficient. This decrease in the filtration coefficient is possibly due to a decrease in the surface area available for filtration [19]. As a urology research group, our main aim was to conduct an animal study to evaluate the changes in nephrons, tubules, and interstitial tissue at different times following obstruction. In our daily practice, we encounter patients who have ureteral stones in various locations (upper, middle, and lower), as well as a severe grade of hydronephrosis, who refuse definite intervention despite experiencing symptoms for a long time. Therefore, having studies that examine the changes in renal tissue after different periods would help advise patients on the optimal time for intervention.

In a study by Andren-Sandberg et al., 358 patients with ureteral stones were evaluated with contrast imaging and excretory urography until the stone had left the ureter. They also followed up with the patients after the release of obstruction with serial imaging and permanent renal damage was found in 24 patients (7%) on an average of 17 months after the passage of the stone. Interesting findings were that the larger stones(>10 mm), advanced age, women, and stones that remained in the ureter for more than three months were associated with enduring damage to renal function [20].

Holm‐Nielsen et al. evaluated 143 individuals suffering from ureteric stones with renography during and after the relief of obstruction and followed them up for four years. They found that the stone size was not correlated with renal function loss. There was no renal function impairment in short-term obstruction (<2 weeks). Infections are proximal to ureteric stones, and age >50 years was associated with accelerated kidney damage [21].

In a preliminary animal study on male rabbits by Johnson et al., they concluded that complete unilateral ureteral obstruction for less than two weeks was not associated with significant renal damage. However, the longer time of the obstruction was associated with more renal damage, especially in the lateral regions of the kidney compared to the medial portions [22].

Pridgen et al., in a canine model study, evaluated the effects of the complete ureteral obstruction in the ipsilateral kidney and the contralateral kidney regarding the duration of the obstruction. The time limit for significant renal damage was defined as four weeks. The glomerular function was affected less by the obstruction than the tubular segments. The more susceptible segments were collecting tubules and distal convoluted tubules, which showed degrees of dilatation and atrophy with cast formation. They also mentioned significant interstitial fibrosis related to the obstruction [23,24].

Our study also revealed the same results as previous studies. Obstruction did not change the glomerular morphology in three intervention groups after six weeks of obstruction.

A study by Miller et al. evaluated seventy-five patients with ureteral stones to determine the most important factors related to the spontaneous stone passage. Their study revealed that a stone size of less than 5 mm, a right-sided stone, and a more distal location were associated with a greater chance of spontaneous stone passage [25].

In an animal study by Ito et al., they demonstrated that the early release of 3-day UUO was associated with a total recovery of RBF and GFR by 14 days after the release of obstruction [26].

An exciting animal study by Vaughan et al. examined the effect of total unilateral ureteral obstruction by occluding the ureter in fifteen female dogs. They studied three groups of dogs with occluded ureters at one, two, and four weeks, respectively. They were followed up for 2, 4, 6, and 20 weeks after the release of the obstruction till sacrificing the dogs at the end of the study. During these periods, the assessment of renal function by blood tests and GFR was performed. Pathologic changes of glomeruli and tubules were also evaluated at the mentioned times. The most significant renal function impairment occurred in the third group, which had four weeks of obstruction. The most frequent pathological changes were observed in collecting duct and distal convoluted tubules. The glomerular changes were the slightest reported abnormalities among the three groups [2].

Obstructive uropathy due to urolithiasis is one of the preventable causes of renal failure, especially in recurrent stone formers. The preliminary animal studies regarding the effects of ureteral obstruction on renal function were mainly based on the experimental occlusion of the ureter in animals such as dogs, rats, rabbits, and mice. These studies primarily focused on imaging, blood tests, and pathologic changes after total UUO. Our analysis also created a model for complete ureteral obstruction, so further studies with a partial obstruction model in different locations of the ureter are needed.

Lucarelli et al. evaluated 76 patients with an obstructive ureteral injury who underwent surgical correction of obstruction within two weeks after obstruction (36 patients) or after two weeks of obstruction (40 patients). Patients who underwent surgery beyond two weeks of obstruction had lower GFR, more radionuclide renal scan changes, and more postoperative hypertension incidence during long-term follow-up [27].

The main aim of our study was to determine the suitable interval of observation before planning any surgical intervention. The main impact of UUO is on the tubulointerstitial component, and nephron loss is less prominent. The cellular and molecular approach to the study of ureteral obstruction may suggest new therapeutic modalities to reduce the impact of inflammatory factors and kidney responses during urinary tract obstruction.

The main pathologic changes to the glomeruli studied in the literature after UUO include glomerular, tubulointerstitial changes, interstitial fibrosis, and infiltration of inflammatory cells. One of the earliest changes is the infiltration of interstitial tissue by inflammatory cells such as macrophages that produce a different kind of cytokines and, consequently, the occurrence of tubulointerstitial or interstitial fibrosis [28–31].

In tubulopathy and interstitial fibrosis secondary to the ureteral obstruction, the production of reactive oxygen species (ROS) in the kidney and an increase in the renal expression of TNF-*α* have a pivotal role. The tubular changes are dilation and eventual tubular atrophy [32–35]. In a study by Guo et al., TNF-*α* receptor type 1 knockout mice had a reduced degree of tubulointerstitial fibrosis following UUO, emphasizing this factor's role in tubular apoptosis [36].

Hammad et al. evaluated the long-term effects of 3-day reversible UUO in a rat model with 18 months' follow-up regarding the GFR and pathologic and physiologic changes. They showed that regarding the recovery of GFR to normal limits, there was ongoing tubulointerstitial fibrosis urinary albumin leakage and alterations in renal injury markers, proinflammatory, and profibrotic cytokines after the reversing of the obstruction [37].

In our study, changes in TNF-*α* were not significantly different between the proximal, mid, and distal ureteral obstruction (*p* value <0.261). However, our study had interesting findings regarding tubulopathy (mainly tubular atrophy). We categorized the severity of tubular atrophy on a qualitative scale, including negative, mild (+), moderate (++), and severe (+++) tubular atrophy. The commencing of severe tubular atrophy in proximal, mid, and distal ureteral obstruction was at weeks three, four, and six, respectively. These results may recommend that the time limit to prevent severe tubulopathy in proximal obstruction is three weeks, whereas this time is extended to six weeks in distal ureteral obstruction. While the control group had higher creatinine levels, all groups showed similar levels of creatinine in the postoperative week 6. Therefore, creatinine levels cannot be relied upon to determine the appropriate time for intervention in obstructed kidneys. It is possible that the changes observed were due to the nature of the animal study or changes in environmental conditions in the control group. Some results were observed in our study including the weight of kidneys after obstruction and the weight of the damaged kidney (at week 6), which were significantly different in the distal ureteral obstruction group (*p* value: 0.046). We believe that the higher grade of hydronephrosis in the distal complete obstruction group might be the only possible reason for these changes. To our knowledge, this study presents the new definition for the first time to determine the optimal time for surgical intervention in the obstructed ureter according to the pathologic findings. This study has some limitations. Firstly, we evaluated the impact of complete ureteral obstruction in different levels of the ureter, but the effect of incomplete ureteral obstruction on renal and tubular damages could not be evaluated; further study with this pattern of obstruction is rational. Secondly, in our study only, the ipsilateral kidney and ureter were assessed; the contralateral changes may also occur in unilateral obstruction. Finally, we only measured one inflammatory factor; a more comprehensive measurement of the inflammatory factors such as interleukin-1 (IL-1), transforming growth factor *β*1 (TGF- *β*1), and fibroblast growth factor (FGF) is recommended to evaluate the most striking factors in different levels of obstruction. This study revealed significant changes in laboratory and clinical findings in the proximal ureteral obstruction group compared to distal obstruction over time, obstructed ureter. However, there is an opportunity to study the effect of the anti-inflammatory and antifibrotic drugs on the different anatomical levels of the ureteral obstruction (upper, middle, and lower ureter) in future studies.

## 5. Conclusion

The location of ureteral obstruction is also crucial in deciding to intervene to relieve the complete ureteral obstruction. Severe tubular damage occurs in weeks three, four, and six in proximal, mid, and distal ureteral obstruction, respectively. The results recommend intervening in the obstruction in the proximal ureter at an earlier time.

## Figures and Tables

**Table 1:** Description of covariates using median (IQR), comparing groups, and times.

	Time after ligation (week)					*p* value
	1	2	3	4	6	
*Urine Cr*						
Group 1	68.9 (68.5–69.3)	73.75 (32.5–115)	87.5 (47–128)	283.5 (166–401)	73 (70–76)	0.406
Group 2	67.25 (56–78.5)	204 (56–352)	97.25 (42.5–152)	78.05 (61.5–94.6)	77.5 (59–96)	1
Group 3	115.75 (19.5–212)	97.75 (73.5–122)	119.25 (95.5–143)	46 (20–72)	53.55 (45.5–61.6)	0.092
Control	32.75 (28.5–37)	131.75 (70.5–193)	91.3 (61.6–121)	46 (20–72)	48.3 (23.1–73.5)	0.406
*p* value	0.261	1	1	0.118	0.261	

*Urea*						
Group 1	51.5 (48–55)	32 (28–36)	50.78 (43.5–58.05)	45.25 (45–45.5)	63.25 (62–64.5)	0.092
Group 2	51 (42.5–59.5)	36.75 (30.5–43)	46.25 (41.5–51)	54 (51.5–56.5)	65 (60–70)	0.092
Group 3	54.5 (36.5–72.5)	50.25 (42.5–58)	53.25 (43–63.5)	51.75 (50.5–53)	50.25 (40–60.5)	1
Control	30.025 (28–32.05)	27 (25–29)	35.75 (33.5–38)	41.75 (38.5–45)	44 (42–46)	0.092
*p* value	0.261	0.261	0.261	0.261	0.261	

*Cr*						
Group 1	0.61 (0.34–0.88)	0.73 (0.71–0.75)	0.945 (0.89–1)	0.93 (0.74–1.12)	0.59 (0.51–0.68)	0.406
Group 2	0.66 (0.49–0.83)	0.795 (0.74–0.85)	1.015 (0.96–1.07)	0.4055 (0.091–0.72)	0.645 (0.53–0.76)	0.406
Group 3	0.475 (0.24–0.71)	0.67 (0.63–0.71)	0.63 (0.58–0.68)	0.55 (0.44–0.66)	0.405 (0.36–0.45)	0.406
Control	0.765 (0.71–0.82)	0.49 (0.46–0.52)	1.02 (0.76–1.28)	0.94 (0.91–0.97)	0.525 (0.43–0.62)	0.092
*p* value	0.659	0.118	0.261	0.046	0.261	

*Na*						
Group 1	144.5 (139–150)	146 (141–151)	139.5 (138–141)	143 (143–143)	147.5 (146–149)	0.212
Group 2	143 (142–144)	145 (144–146)	147.5 (144–151)	141 (140–142)	147.5 (147–148)	0.212
Group 3	148.5 (147–150)	146 (143–149)	141 (138–144)	146 (142–150)	143.5 (139–148)	0.406
Control	148.5 (146–151)	163 (161–165)	143.5 (138–149)	142.5 (140–145)	150.5 (150–151)	0.092
*p* value	0.261	0.261	0.261	0.261	0.118	

*K*						
Group 1	4.1 (4.01–4.19)	4.495 (4.08–4.91)	4.195 (4.07–4.32)	4.32 (4.28–4.36)	4.36 (4.33–4.39)	0.406
Group 2	4.185 (4.11–4.26)	4.37 (4.18–4.56)	4.32 (4.111–4.52)	4.48 (4.19–4.77)	4.525 (4.19–4.86)	1
Group 3	4.565 (4.27–4.86)	4.935 (4.76–5.11)	4.33 (4.27–4.39)	5.145 (5.1–5.19)	4.665 (4.33–5)	0.406
Control	4.6 (4.29–4.91)	4.55 (4.09–5.01)	4.815 (4.62–5.01)	4.23 (4.19–4.27)	4.62 (4.28–4.96)	0.406
*p* value	0.046	0.261	0.261	0.261	1	

	Times					*p* value
	1	2	3	4	6	

*TNF*						
Group 1	11.495 (10.32–12.67)	16.935 (10.02–23.85)	15.535 (11.05–20.02)	8.845 (3.55–14.14)	12.67 (9.14–16.2)	1
Group 2	12.085 (6.2–17.97)	18.555 (16.79–20.32)	19.44 (17.38–21.5)	8.1155 (5.911–10.32)	10.61 (5.61–15.61)	0.092
Group 3	10.76 (5.61–15.91)	10.32 (6.79–13.85)	9.585 (8.85–10.32)	12.675 (12.38–12.97)	16.35 (16.2–16.5)	0.406
Control	19.85 (15.85–23.85)	17.46 (16.61–18.31)	20.76 (19.14–22.38)	10.3205 (5.911–14.73)	12.375 (11.2–13.55)	0.092
*p* value	0.261	0.261	0.261	0.261	0.261	

*Weight before*						
Group 1	159.5 (153.9–165.1)	177.95 (176.1–179.8)	201 (200–202)	201.9 (198.2–205.6)	180.2 (162–198.4)	0.092
Group 2	179.4 (159.5–199.3)	200.3 (200–200.6)	199.6 (199.2–200)	198.3 (198–198.6)	184.5 (168–201)	0.406
Group 3	199.85 (199–200.7)	197.75 (197.3–198.2)	193.85 (189.2–198.5)	192 (184–200)	199.7 (199.2–200.2)	0.092
Control	191.3 (184–198.6)	192.15 (186.9–197.4)	195.7 (189.8–201.6)	195.8 (187.9–203.7)	199.9 (199.8–200)	0.406
*p* value	0.261	0.261	0.261	1	0.261	

*Weight after*						
Group 1	161.65 (155.8–167.5)	183.65 (183–184.3)	209.4 (206.9–211.9)	212.25 (204–220.5)	201.7 (184.2–219.2)	0.092
Group 2	182.45 (161.1–203.8)	221.8 (218.6–225)	216 (205.7–226.3)	209.8 (205.7–213.9)	212.3 (187.6–237)	0.406
Group 3	228 (220–236)	224.5 (220–229)	204.4 (198.6–210.2)	205.25 (199.6–210.9)	220.5 (205.7–235.3)	0.092
Control	202.7 (199.6–205.8)	205.95 (202.3–209.6)	210.95 (207.6–214.3)	209.05 (207.5–210.6)	211.45 (208.5–214.4)	0.406
*p* value	0.261	0.046	1	1	1	

*Temperature*						
Group 1	35.65 (35.6–35.7)	35.7 (35.6–35.8)	35.65 (35.6–35.7)	35.75 (35.7–35.8)	35.65 (35.6–35.7)	0.441
Group 2	35.8 (35.6–36)	35.85 (35.7–36)	35.65 (35.6–35.7)	35.8 (35.8–35.8)	35.45 (35.4–35.5)	0.212
Group 3	36 (36–36)	35.9 (35.8–36)	35.65 (35.6–35.7)	35.75 (35.7–35.8)	35.8 (35.6–36)	0.212
Control	35.85 (35.8–35.9)	35.6 (35.5–35.7)	35.7 (35.4–36)	35.65 (35.6–35.7)	35.7 (35.7–35.7)	0.107
*p* value	0.261	0.261	1	0.261	0.261	

*Weight of damaged kidney*						
Group 1	3.15 (2.6–3.7)	4.85 (4.2–5.5)	5.85 (5.8–5.9)	5.95 (5.8–6.1)	5.95 (5.9–6)	0.212
Group 2	2.6 (2.4–2.8)	4.4 (2.5–6.3)	5.5 (5.4–5.6)	6.6 (6.4–6.8)	6.55 (6.5–6.6)	0.092
Group 3	4.8 (4.6–5)	5.5 (5.4–5.6)	5.85 (5.8–5.9)	6.35 (6.1–6.6)	6.85 (6.8–6.9)	0.092
Control	3.6 (3.5–3.7)	3.95 (3.9–4)	4.55 (4.3–4.8)	5.1 (5–5.2)	6.1 (6–6.2)	0.092
*p* value	0.261	0.261	0.046	0.118	0.046	

**Table 2:** Proximal ureteral obstruction.

Weeks after obstruction	Tubular atrophy	Interstitial fibrosis	Interstitial inflammation (%)
1	+	−	10–20
2	++	+	10–20
3	+++	++	50
4	+++	+++	80
6	+++	+++	90

−Negative/+ mild/++ moderate/+++ severe.

**Table 3:** Ureteral obstruction.

Weeks after obstruction	Tubular atrophy	Interstitial fibrosis	Interstitial inflammation (%)
1	−	−	10
2	+	+	30
3	++	++	50
4	+++	+++	80
6	+++	+++	90

−Negative/+ mild/++ moderate/+++ severe.

**Table 4:** Distal ureteral obstruction.

Weeks after obstruction	Tubular atrophy	Interstitial fibrosis	Interstitial inflammation (%)
1	−	−	10
2	−	+	30
3	+	++	50
4	++	+++	70
6	+++	+++	90

−Negative/+ mild/++ moderate/+++ severe.

## Data Availability

All information, data, and photos are provided through the manuscript, and additional data will be provided if requested. The corresponding author is responsible for the data availability if needed.

## Supplementary Materials

Supplementary Materials Ultrasonic pictures of three different groups of ureteral ligation in weeks 1, 2, 4, and 6.
