# Supplementary figures and images for: Effects of the Surgical Ligation of the Ureter in Different Locations on the Kidney over Time in the Rat Model
Source: Adv Urol. 2024 Jun 6;2024:6611081. doi: 10.1155/2024/6611081 (PMC11221982; doi:10.1155/2024/6611081)

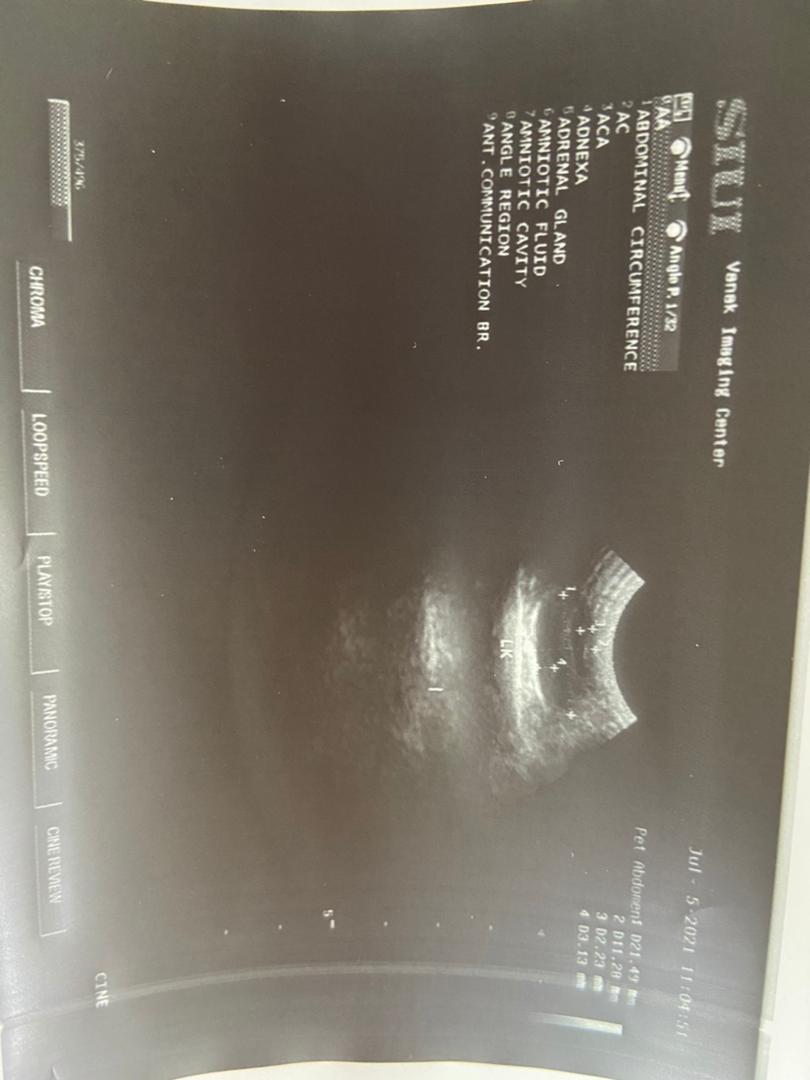

Supplement: Supplementary Materials — Ultrasonic pictures of three different groups of ureteral ligation in weeks 1, 2, 4, and 6. [file 6611081.f1.zip › WhatsApp Image 2022-02-07 at 11.58.28 (1).jpeg]

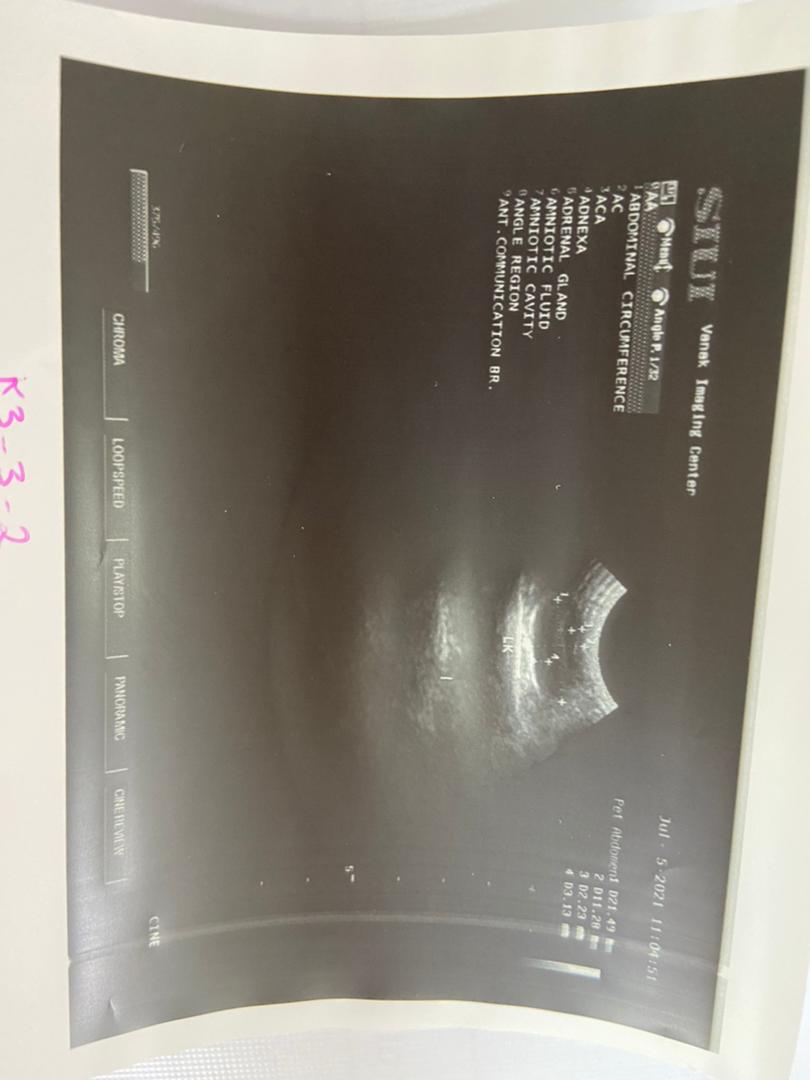

Supplement: Supplementary Materials — Ultrasonic pictures of three different groups of ureteral ligation in weeks 1, 2, 4, and 6. [file 6611081.f1.zip › WhatsApp Image 2022-02-07 at 11.58.28.jpeg]

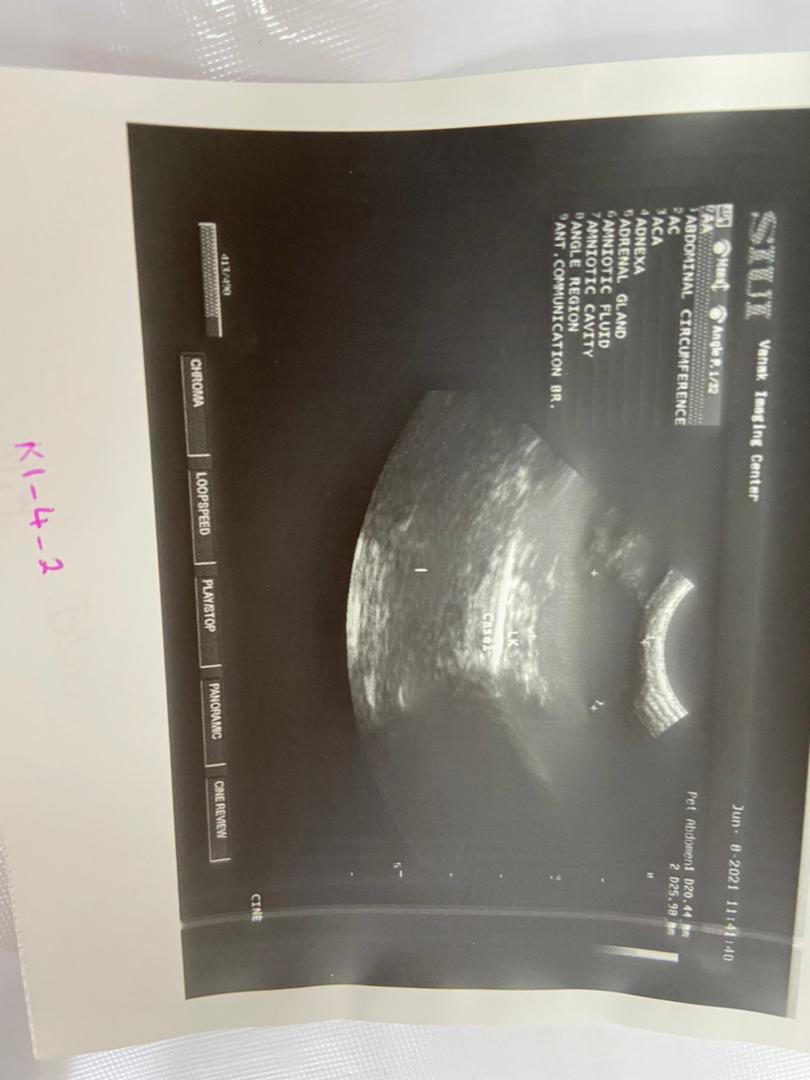

Supplement: Supplementary Materials — Ultrasonic pictures of three different groups of ureteral ligation in weeks 1, 2, 4, and 6. [file 6611081.f1.zip › WhatsApp Image 2022-02-07 at 11.58.29 (1).jpeg]

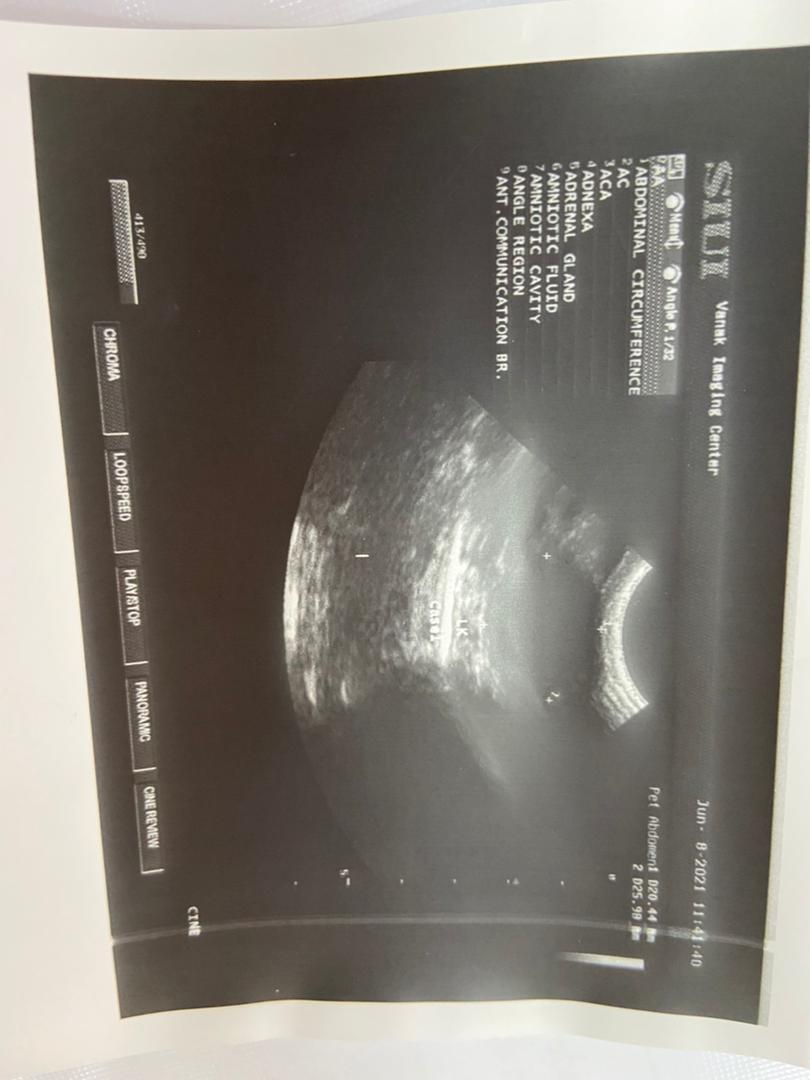

Supplement: Supplementary Materials — Ultrasonic pictures of three different groups of ureteral ligation in weeks 1, 2, 4, and 6. [file 6611081.f1.zip › WhatsApp Image 2022-02-07 at 11.58.29.jpeg]

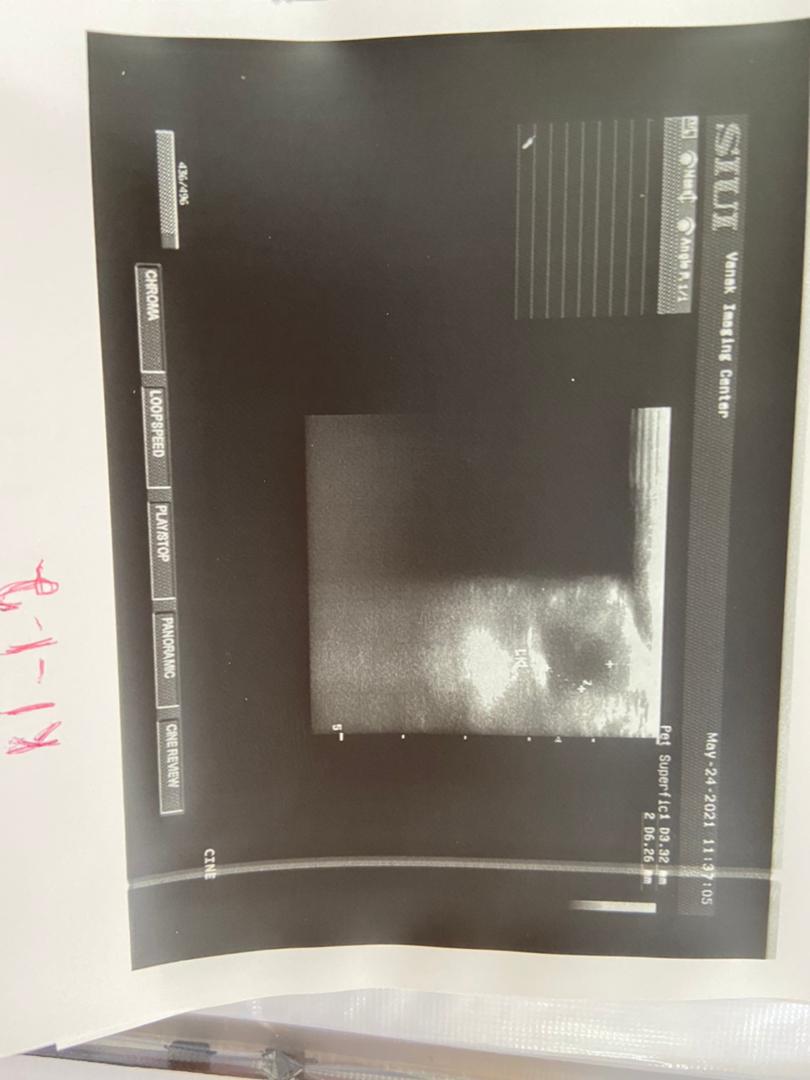

Supplement: Supplementary Materials — Ultrasonic pictures of three different groups of ureteral ligation in weeks 1, 2, 4, and 6. [file 6611081.f1.zip › WhatsApp Image 2022-02-07 at 11.58.30 (1).jpeg]

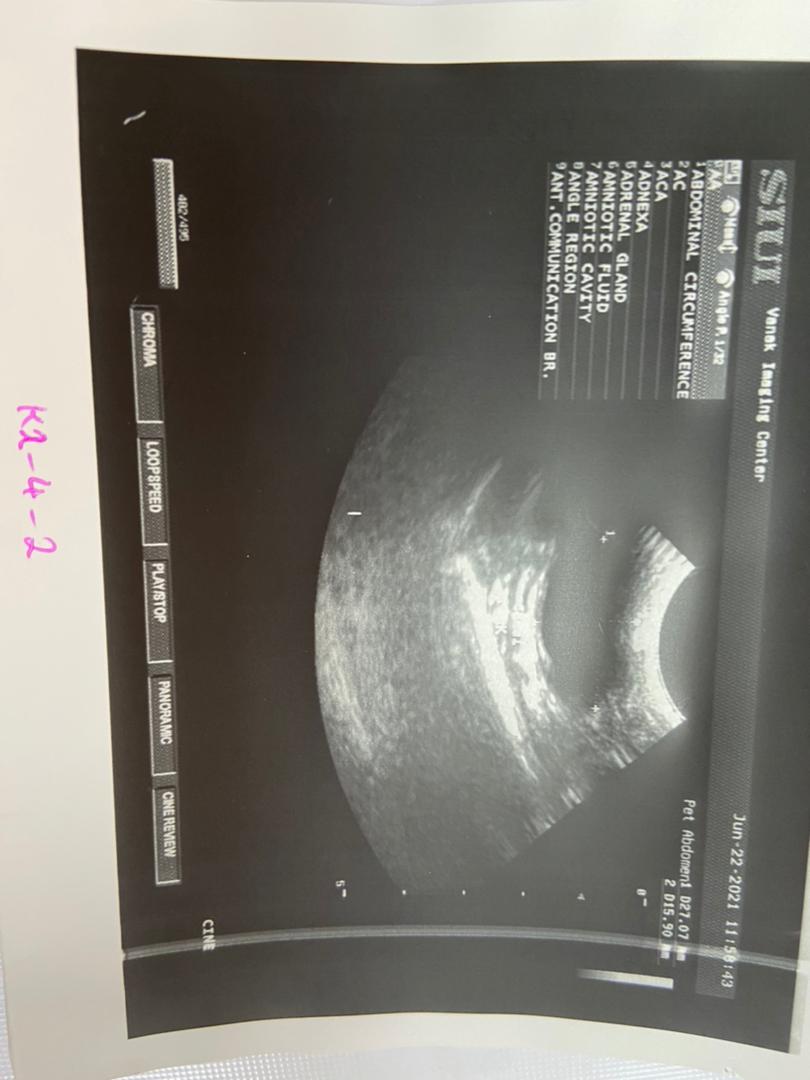

Supplement: Supplementary Materials — Ultrasonic pictures of three different groups of ureteral ligation in weeks 1, 2, 4, and 6. [file 6611081.f1.zip › WhatsApp Image 2022-02-07 at 11.58.30.jpeg]

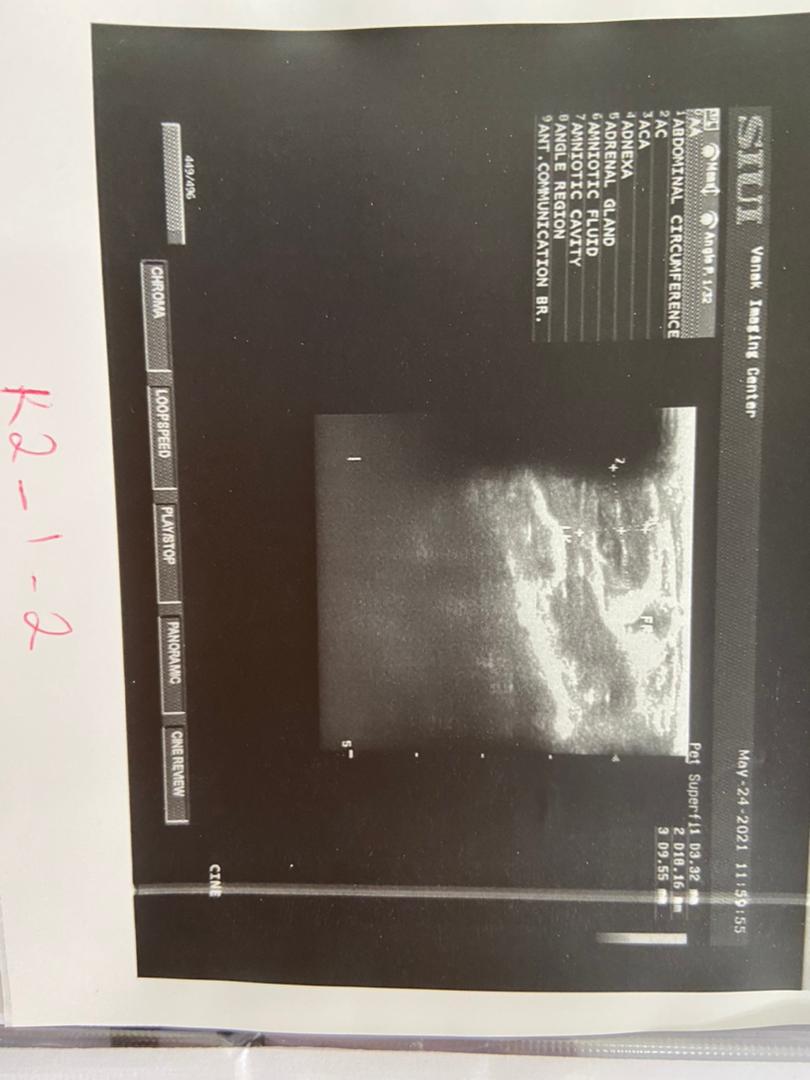

Supplement: Supplementary Materials — Ultrasonic pictures of three different groups of ureteral ligation in weeks 1, 2, 4, and 6. [file 6611081.f1.zip › WhatsApp Image 2022-02-07 at 11.58.31 (1).jpeg]

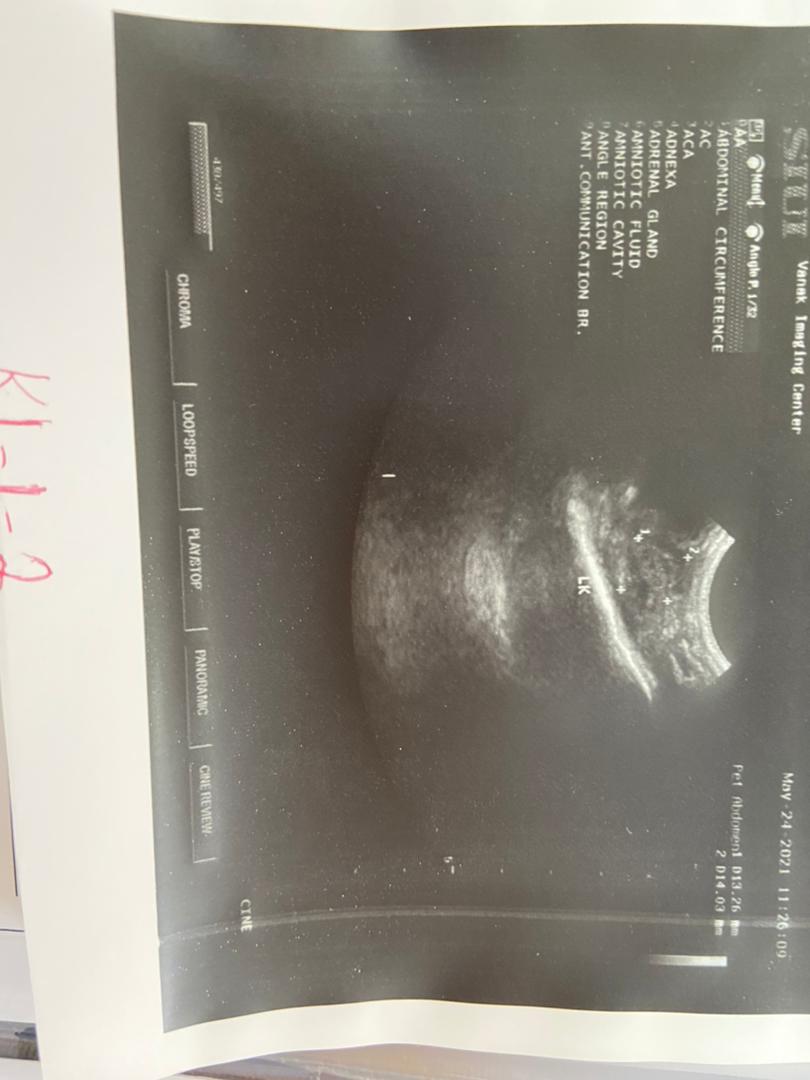

Supplement: Supplementary Materials — Ultrasonic pictures of three different groups of ureteral ligation in weeks 1, 2, 4, and 6. [file 6611081.f1.zip › WhatsApp Image 2022-02-07 at 11.58.31.jpeg]

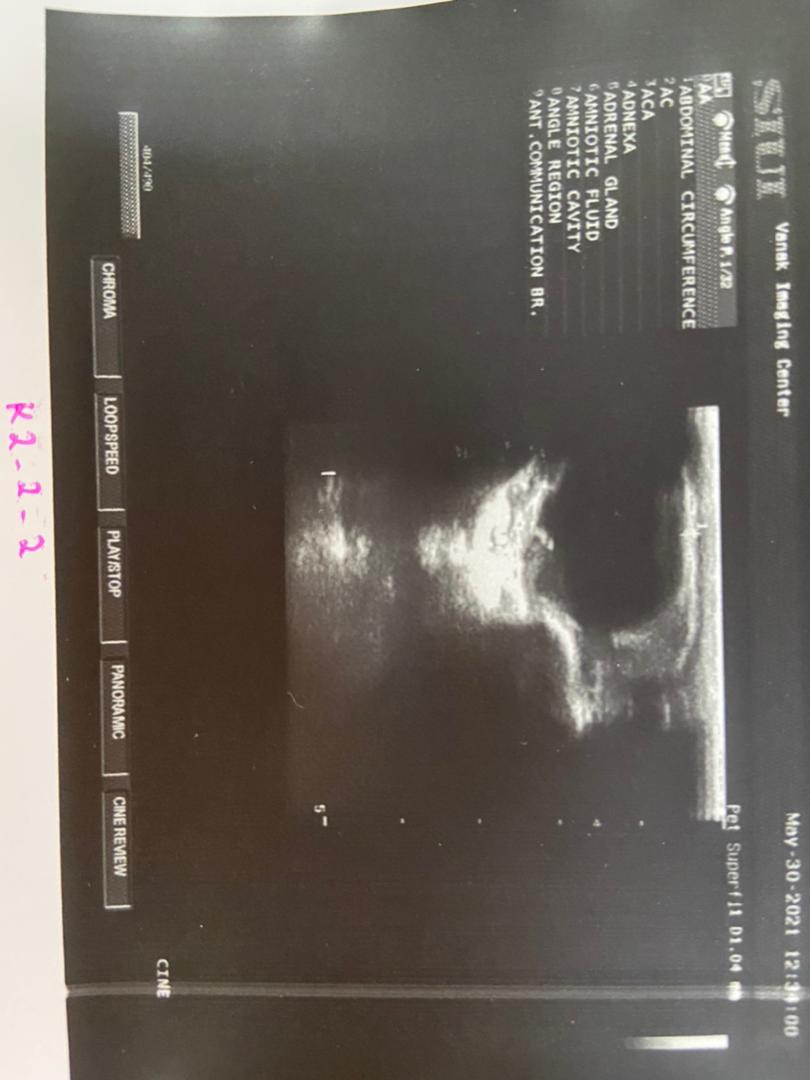

Supplement: Supplementary Materials — Ultrasonic pictures of three different groups of ureteral ligation in weeks 1, 2, 4, and 6. [file 6611081.f1.zip › WhatsApp Image 2022-02-07 at 11.58.32 (1).jpeg]

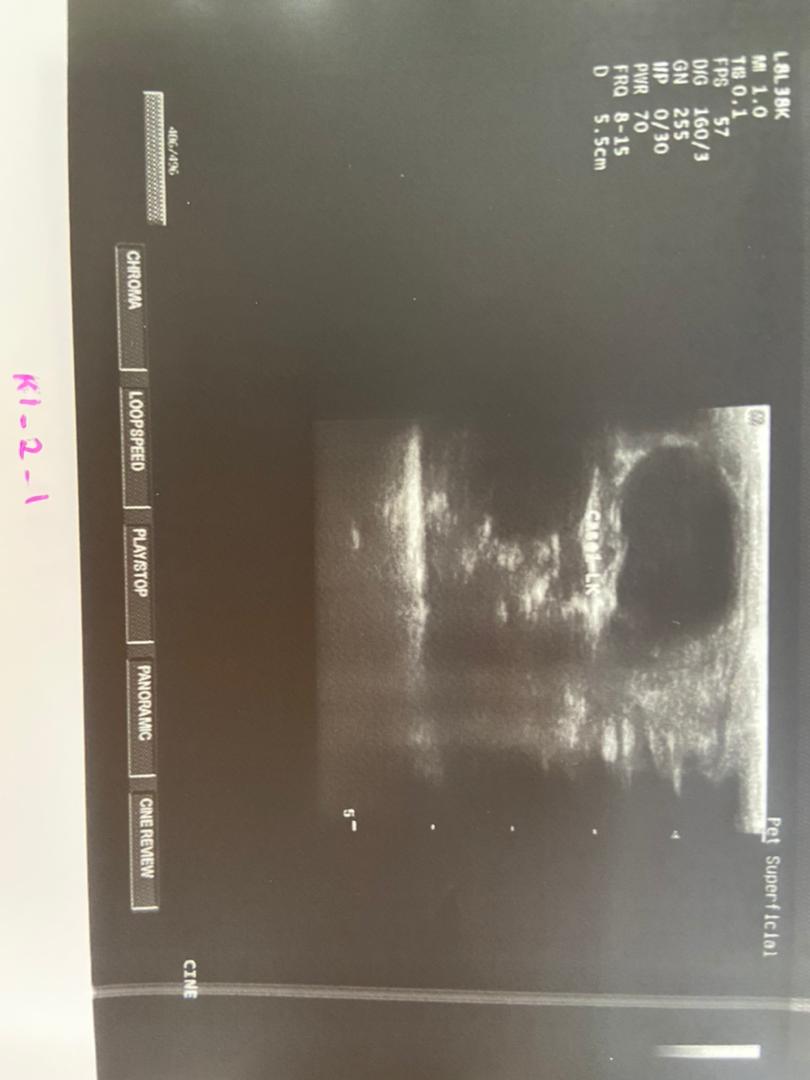

Supplement: Supplementary Materials — Ultrasonic pictures of three different groups of ureteral ligation in weeks 1, 2, 4, and 6. [file 6611081.f1.zip › WhatsApp Image 2022-02-07 at 11.58.32.jpeg]

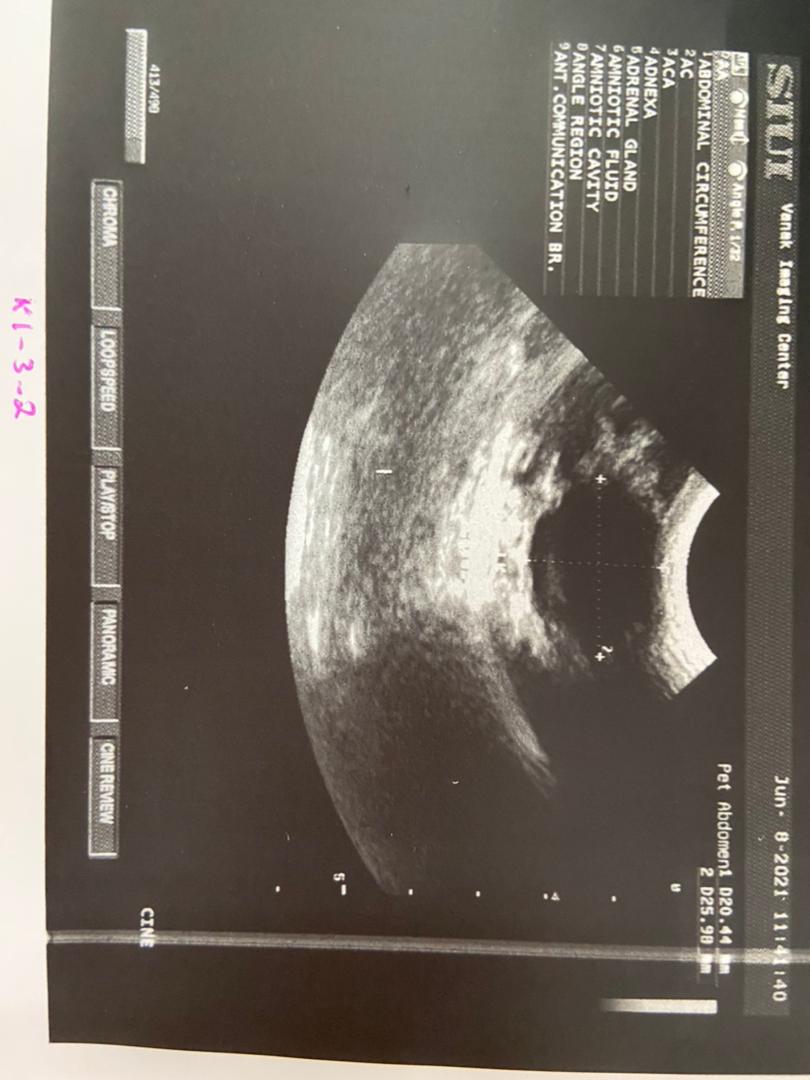

Supplement: Supplementary Materials — Ultrasonic pictures of three different groups of ureteral ligation in weeks 1, 2, 4, and 6. [file 6611081.f1.zip › WhatsApp Image 2022-02-07 at 11.58.33 (1).jpeg]

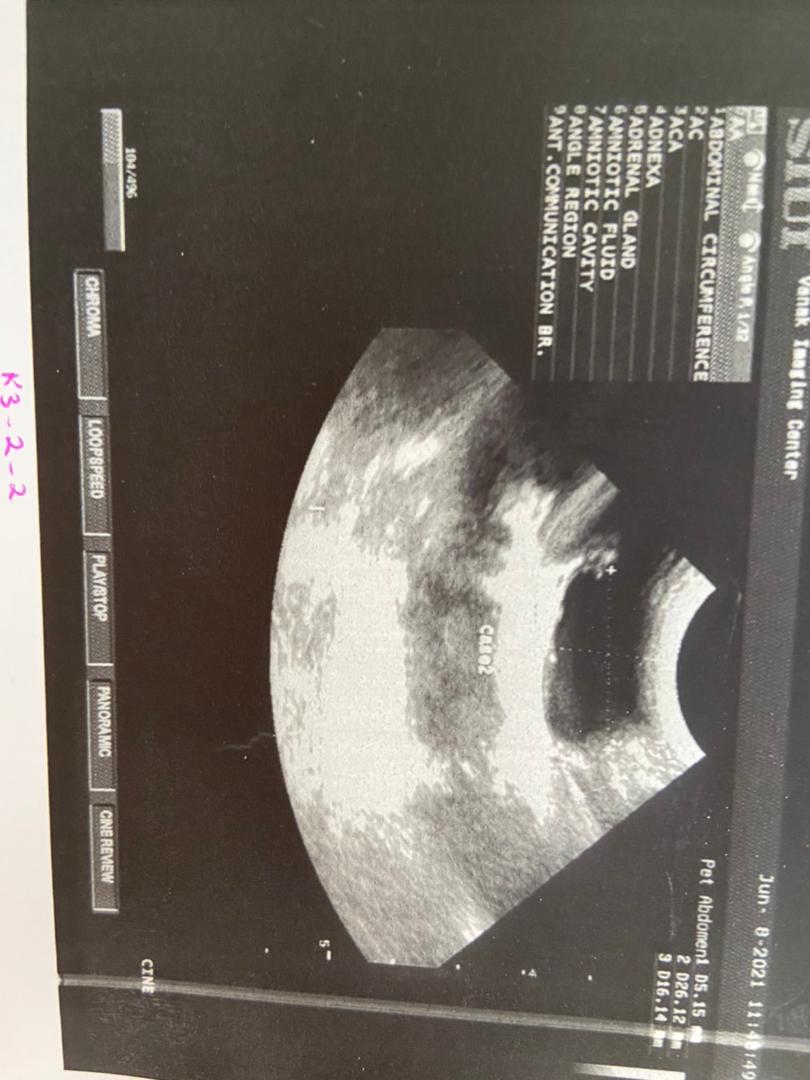

Supplement: Supplementary Materials — Ultrasonic pictures of three different groups of ureteral ligation in weeks 1, 2, 4, and 6. [file 6611081.f1.zip › WhatsApp Image 2022-02-07 at 11.58.34 (1).jpeg]

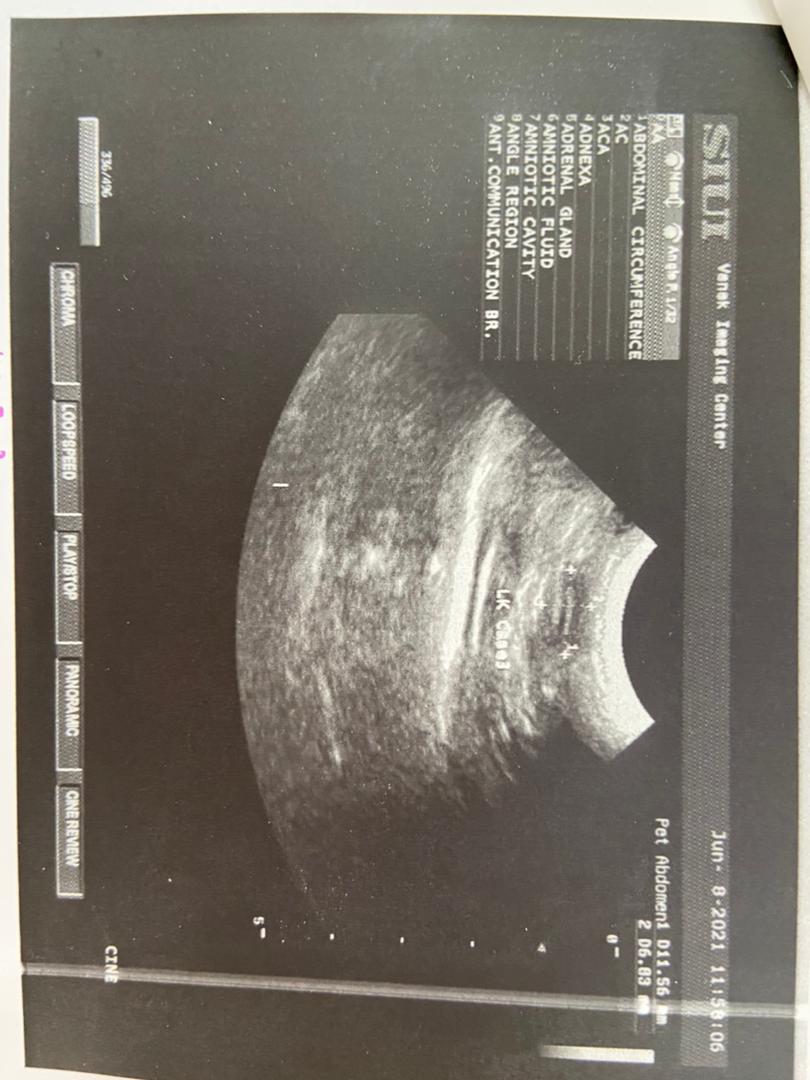

Supplement: Supplementary Materials — Ultrasonic pictures of three different groups of ureteral ligation in weeks 1, 2, 4, and 6. [file 6611081.f1.zip › WhatsApp Image 2022-02-07 at 11.58.34.jpeg]

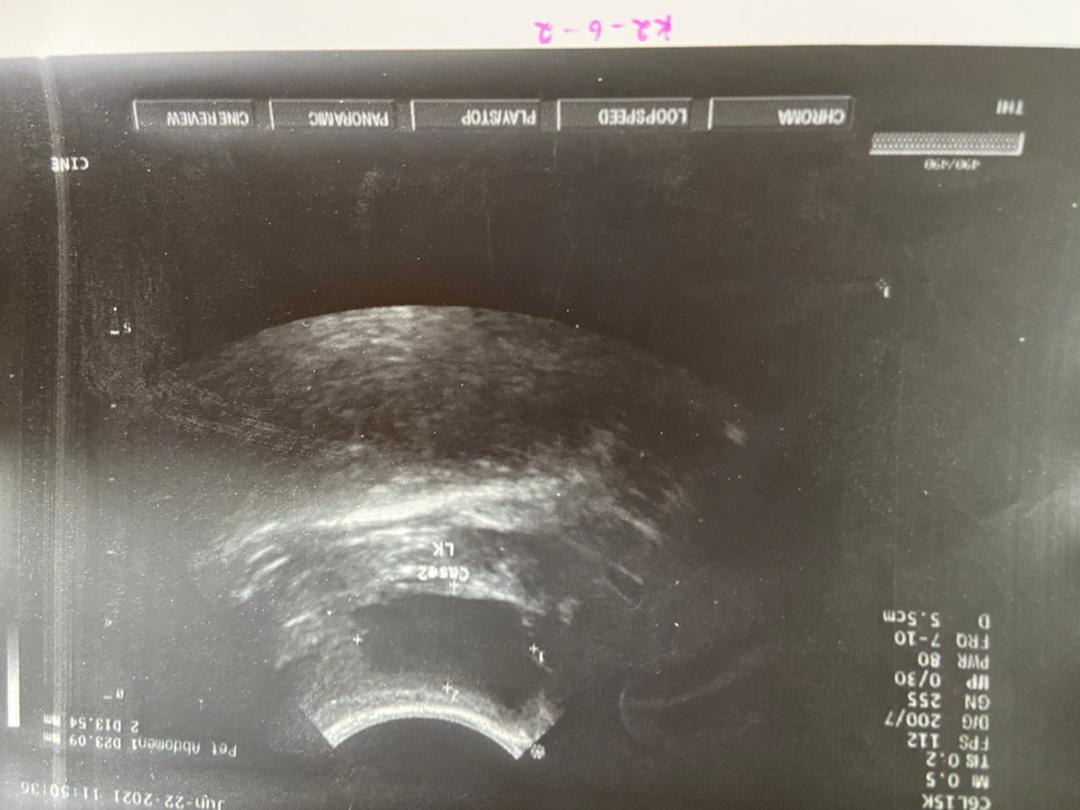

Supplement: Supplementary Materials — Ultrasonic pictures of three different groups of ureteral ligation in weeks 1, 2, 4, and 6. [file 6611081.f1.zip › WhatsApp Image 2022-02-07 at 11.58.35 (1).jpeg]

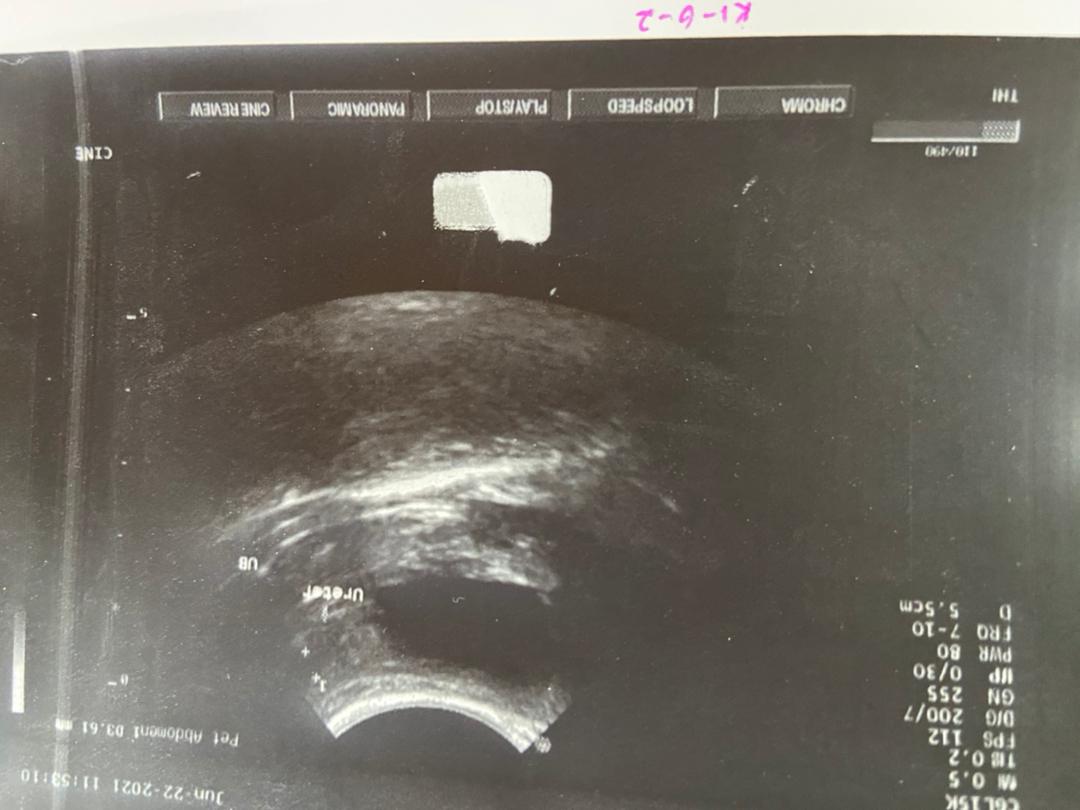

Supplement: Supplementary Materials — Ultrasonic pictures of three different groups of ureteral ligation in weeks 1, 2, 4, and 6. [file 6611081.f1.zip › WhatsApp Image 2022-02-07 at 11.58.35.jpeg]

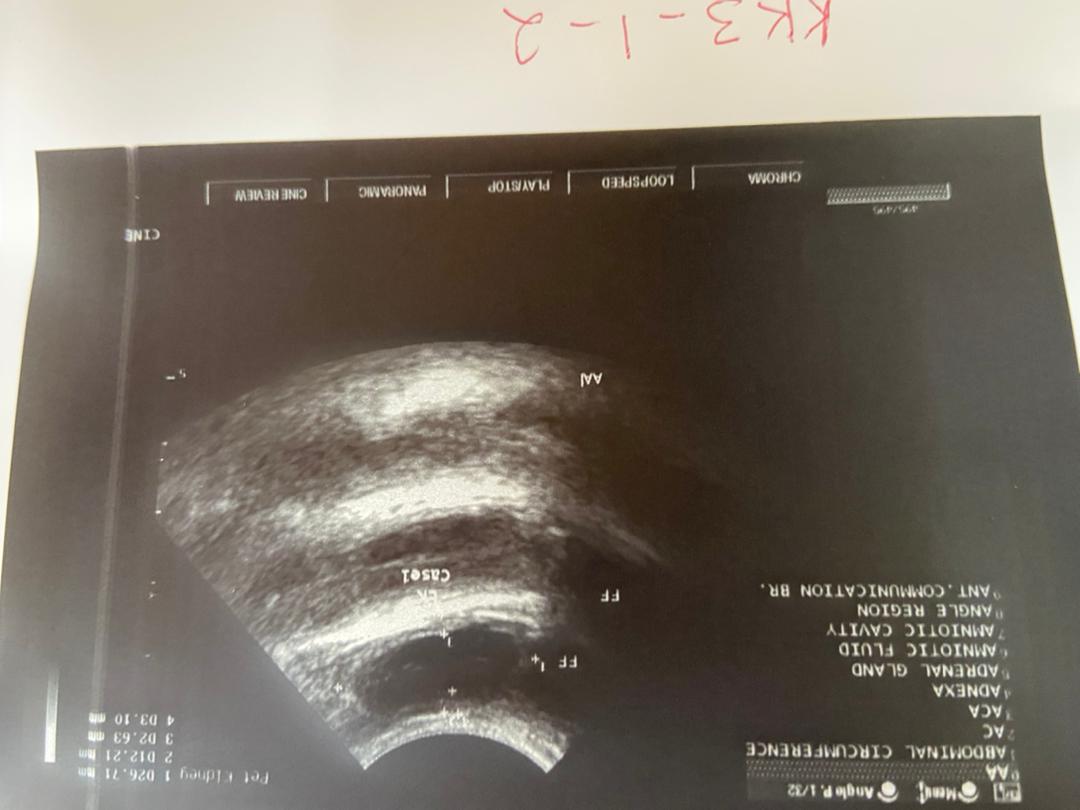

Supplement: Supplementary Materials — Ultrasonic pictures of three different groups of ureteral ligation in weeks 1, 2, 4, and 6. [file 6611081.f1.zip › WhatsApp Image 2022-02-07 at 11.58.36 (1).jpeg]

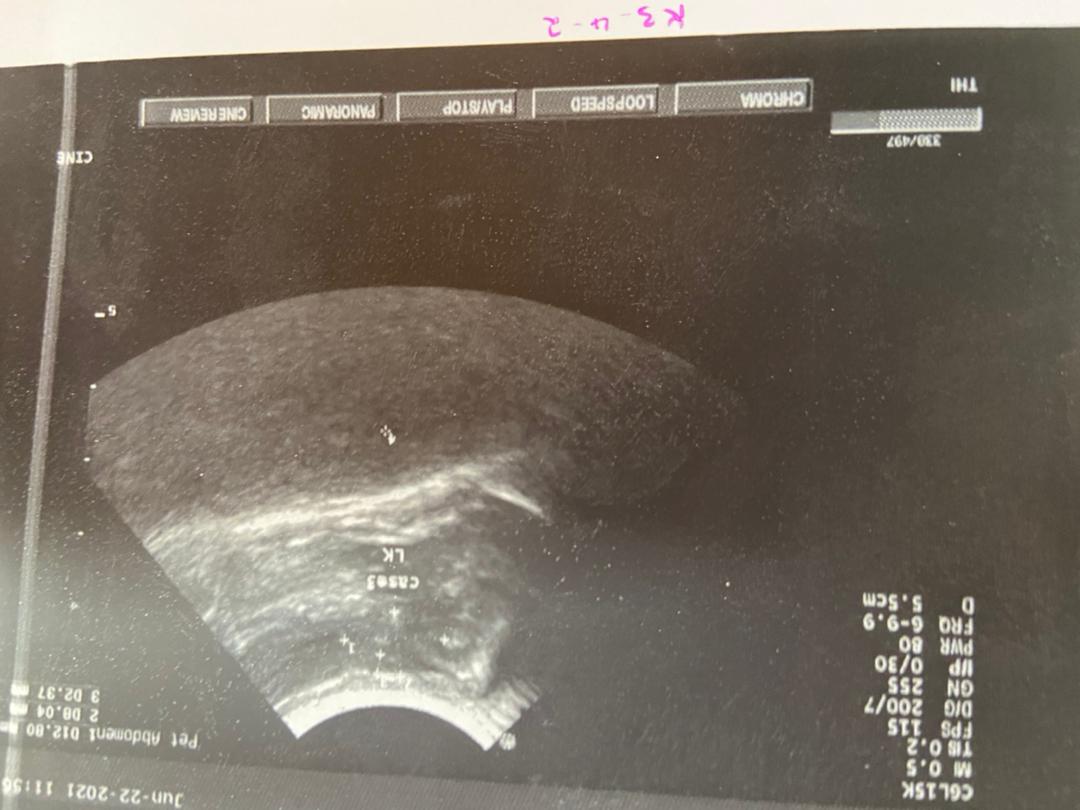

Supplement: Supplementary Materials — Ultrasonic pictures of three different groups of ureteral ligation in weeks 1, 2, 4, and 6. [file 6611081.f1.zip › WhatsApp Image 2022-02-07 at 11.58.36.jpeg]

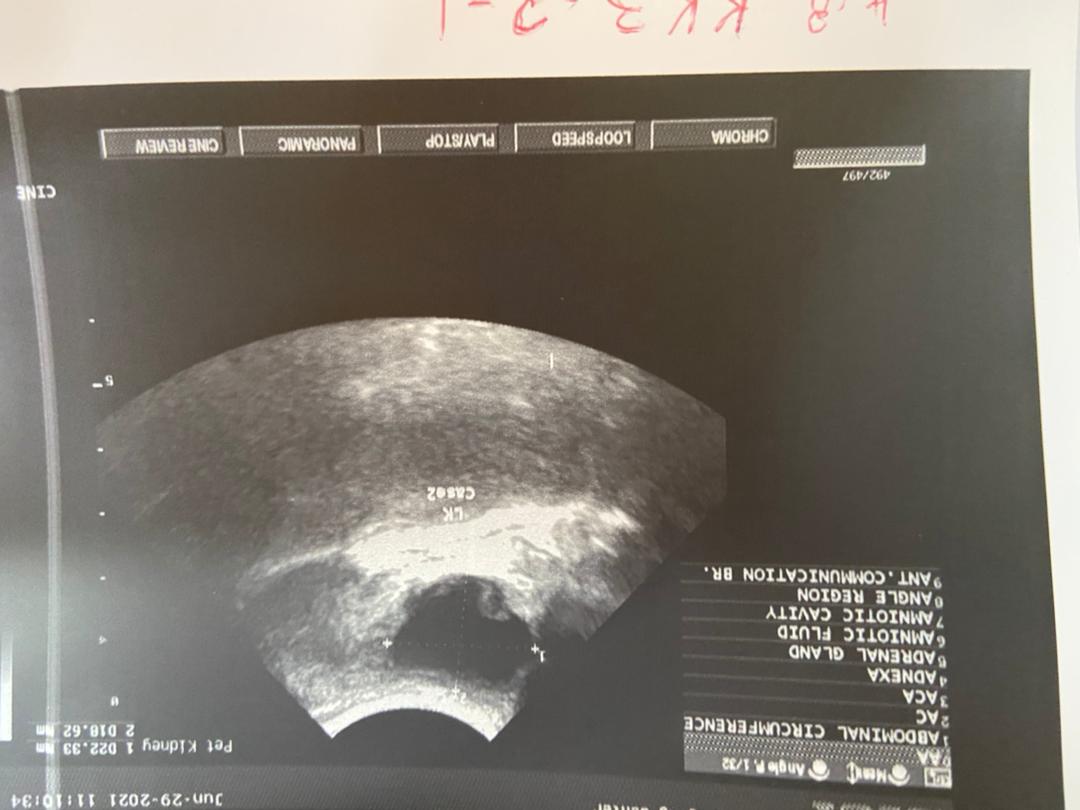

Supplement: Supplementary Materials — Ultrasonic pictures of three different groups of ureteral ligation in weeks 1, 2, 4, and 6. [file 6611081.f1.zip › WhatsApp Image 2022-02-07 at 11.58.37.jpeg]

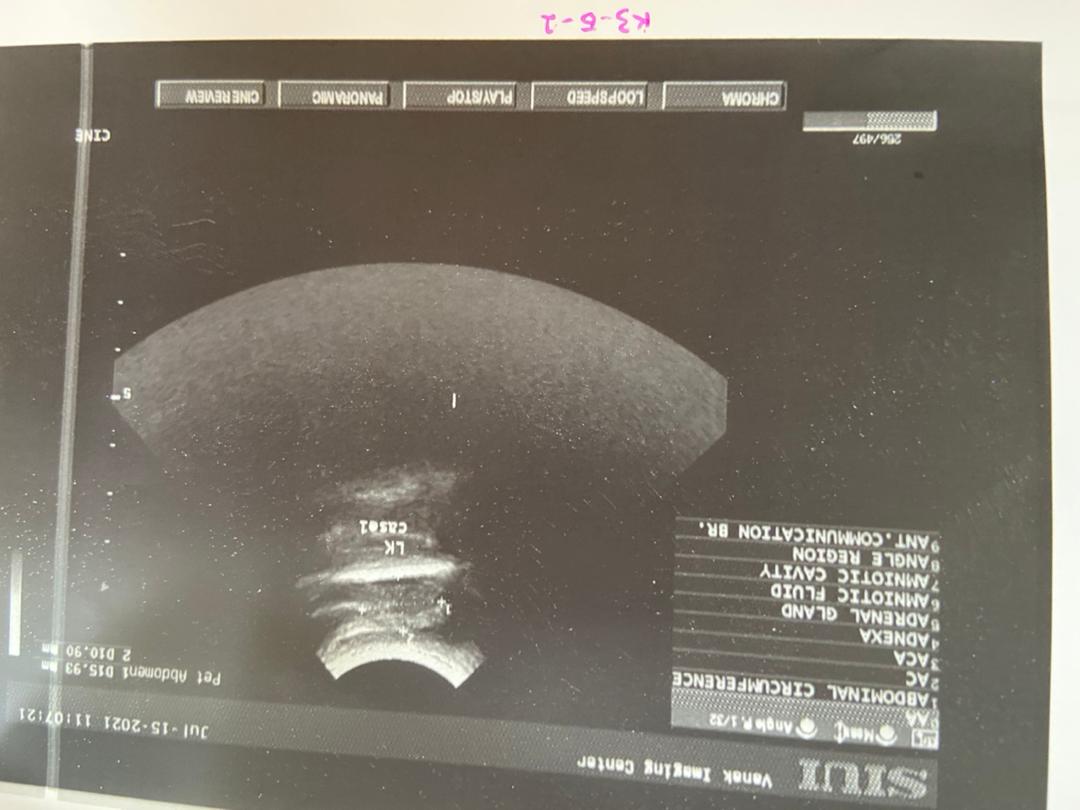

Supplement: Supplementary Materials — Ultrasonic pictures of three different groups of ureteral ligation in weeks 1, 2, 4, and 6. [file 6611081.f1.zip › WhatsApp Image 2022-02-07 at 11.58.38 (1).jpeg]

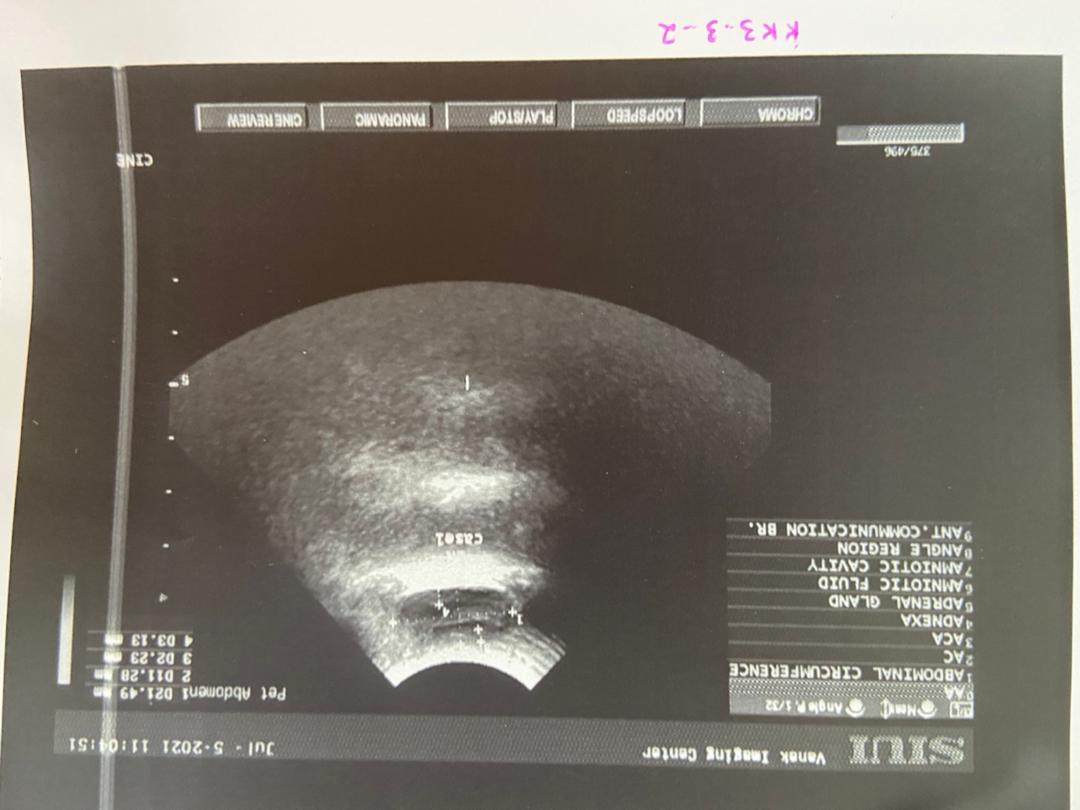

Supplement: Supplementary Materials — Ultrasonic pictures of three different groups of ureteral ligation in weeks 1, 2, 4, and 6. [file 6611081.f1.zip › WhatsApp Image 2022-02-07 at 11.58.38.jpeg]

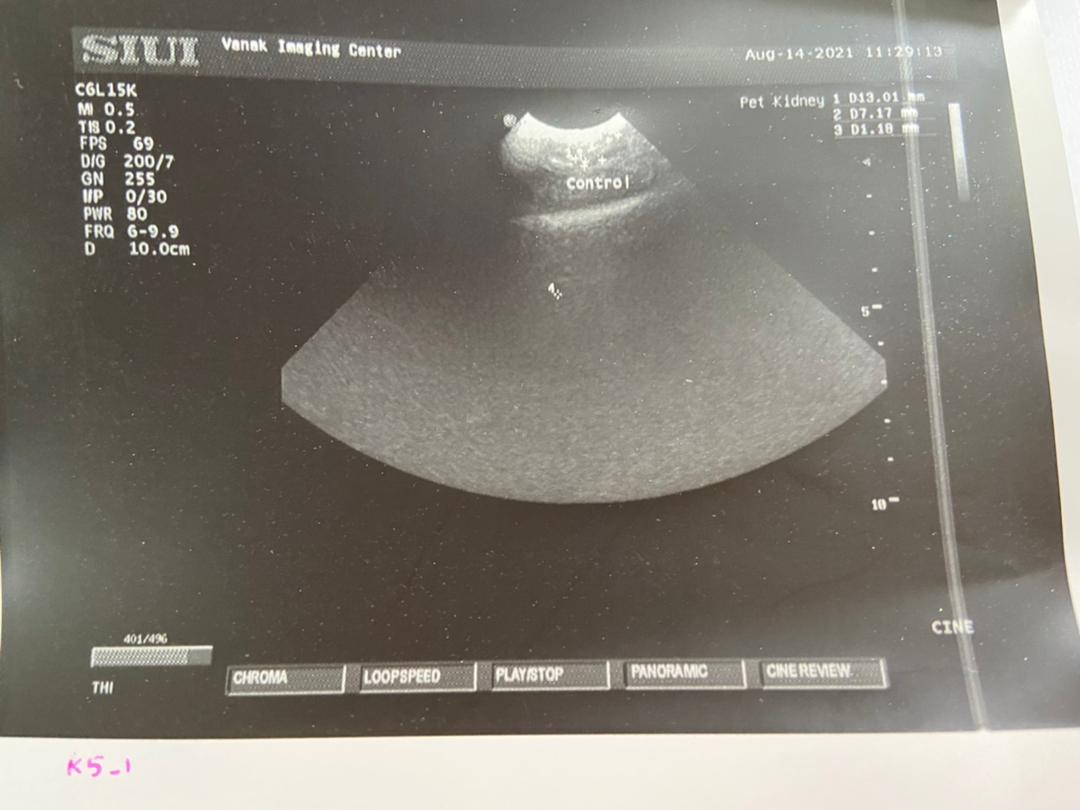

Supplement: Supplementary Materials — Ultrasonic pictures of three different groups of ureteral ligation in weeks 1, 2, 4, and 6. [file 6611081.f1.zip › WhatsApp Image 2022-02-07 at 11.58.39 (1).jpeg]

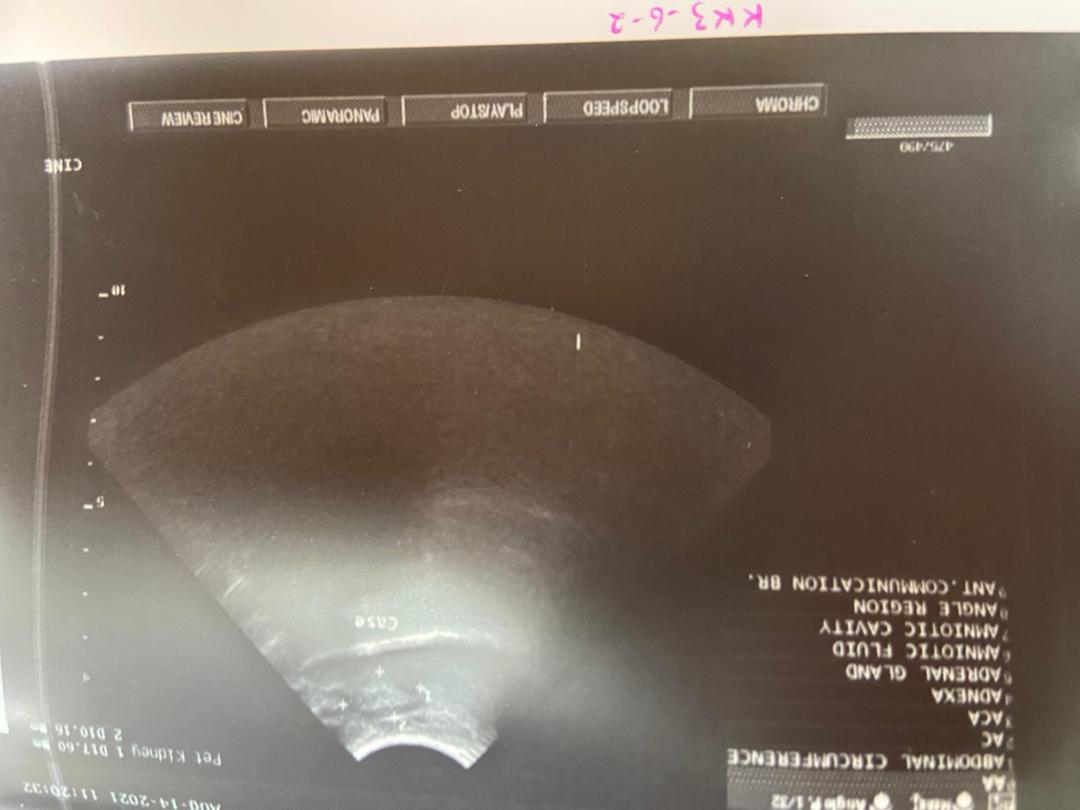

Supplement: Supplementary Materials — Ultrasonic pictures of three different groups of ureteral ligation in weeks 1, 2, 4, and 6. [file 6611081.f1.zip › WhatsApp Image 2022-02-07 at 11.58.39.jpeg]
